# Hierarchical Fe_2_O_3_ hexagonal nanoplatelets anchored on SnO_2_ nanofibers for high-performance asymmetric supercapacitor device

**DOI:** 10.1038/s41598-022-18840-2

**Published:** 2022-09-02

**Authors:** Morteza Safari, Jamal Mazloom, Komail Boustani, Ali Monemdjou

**Affiliations:** 1 Department of Physics, Faculty of Science, University of Guilan, Namjoo Avenue, P.O. Box 4193833697, Rasht, Iran; 2 Department of Physics, University of Science and Technology of Mazandaran, P.O. Box 48518-78195, Behshahr, Iran

**Keywords:** Physics, Applied physics, Condensed-matter physics, Nanoscale materials, Materials science, Electrochemistry

## Abstract

Metal oxide heterostructures have gained huge attention in the energy storage applications due to their outstanding properties compared to pristine metal oxides. Herein, magnetic Fe_2_O_3_@SnO_2_ heterostructures were synthesized by the sol–gel electrospinning method at calcination temperatures of 450 and 600 °C. XRD line profile analysis indicated that fraction of tetragonal tin oxide phase compared to rhombohedral hematite was enhanced by increasing calcination temperature. FESEM images revealed that hexagonal nanoplatelets of Fe_2_O_3_ were hierarchically anchored on SnO_2_ hollow nanofibers. Optical band gap of heterogeneous structures was increased from 2.06 to 2.40 eV by calcination process. Vibrating sample magnetometer analysis demonstrated that increasing calcination temperature of the samples reduces saturation magnetization from 2.32 to 0.92 emu g^-1^. The Fe_2_O_3_@SnO_2_-450 and Fe_2_O_3_@SnO_2_-600 nanofibers as active materials coated onto Ni foams (NF) and their electrochemical performance were evaluated in three and two-electrode configurations in 3 M KOH electrolyte solution. Fe_2_O_3_@SnO_2_-600/NF electrode exhibits a high specific capacitance of 562.3 F g^-1^ at a current density of 1 A g^-1^ and good cycling stability with 92.8% capacitance retention at a high current density of 10 A g^-1^ after 3000 cycles in three-electrode system. The assembled Fe_2_O_3_@SnO_2_-600//activated carbon asymmetric supercapacitor device delivers a maximum energy density of 50.2 Wh kg^-1^ at a power density of 650 W kg^-1^. The results display that the Fe_2_O_3_@SnO_2_-600 can be a promising electrode material in supercapacitor applications.

## Introduction

The growing concerns about the consumption of fossil fuels, environmental pollution and their replacement by the development of clean, efficient electricity sources have drawn more attention to the advanced energy storage devices^1^. Supercapacitors are one of the most importantly researched energy storage devices; depending on the electrode materials can bridge the gap between rechargeable batteries and traditional dielectric capacitors due to their unique properties like long-term cycling stability, high power density, safety and low maintenance cost depending on electrode materials^2^. Over the past few decades, nanostructured materials with governable morphology and dimensions are designed and ready, which totally different functions are achieved by cutting the form, composition, and assembled structure^3^. In particular, one-dimensional (1D) nanomaterials such as nanorods^4^, nanowires^5^, nanotubes^3^, nanofibers^6^, and hollow structures^7^ possess superior properties like high aspect ratio, small dimension structure and unique device function compared to their microscale and bulk counterparts^8^. Moreover, semiconductor nanoheterostructures have attracted a lot of interest due to their optoelectrical^9^, optical^10^, photocatalytic^11^ and electrochemical properties^12^ can be largely improved or amended. Some fantastic characteristics of the heterostructures, like tailoring bandgap, the photo-absorption optimization, structural flexibility and charge carrier mobility enhancement, might give multiple synergistic functions to unravel the problems in environment and energy fields. Semiconductor nanoheterostructures are promising electrode materials for high-rate supercapacitors^13^. Tubular nanomaterials have received significant study attention in recent years due to their hollow shape, which provides a number of unique features that should be beneficial in a variety of applications such as information storage medium^14^, catalysts^15^, electronics^16^, gas sensors^17^, and medicine^18^. Therefore, many methods have been reported for preparing tubular magnetic nanomaterials, including template-assisted electrodeposition^19^, co-precipitation^20^, sol-gel^21^ and solvothermal^22^ methods. Nonetheless, these methods are frequently hampered by time-consuming procedures and special situations. Fundamental research considers the development of simple and effective methods for producing tubular nanostructures at cheap cost to be a significant issue for future practical applications^23^. Transition metal oxides such as Co_3_O_4_, Fe_2_O_3_, and SnO_2_ can offer excellent specific capacity because of the various element valence states of their reversible reactions; as a result, transition metal oxide electrode materials with excellent pseudocapacitance properties are being developed by researchers^24,25^.

Hematite (α-Fe_2_O_3_) material has been considered as a promising material for application in electrochemical energy storage devices due to their non-toxicity, high ideal theoretical capacitance (3625 F g^-1^ in Δ*V* = 1 V)^26^ and abundant reserves. Nevertheless, the low conductivity of Fe_2_O_3_ (~ 10^−14^ S cm^-1^) severely limits its further growth in the energy storage fields and its actual capacitance (120–320 F g^-1^) is very low compared to the theoretical value^27,28^. Many efforts have been taken to address this issue, including the development of Fe_2_O_3_-based composites, nanostructured Fe_2_O_3_ and oxygen-deficient Fe_2_O_3_. The most operative of these strategies is to build Fe_2_O_3_-based composites by utilizing the synergistic effect among different materials^25,29^. Furthermore, SnO_2_ nanomaterials are one of the most important typical *n*-type metal oxide semiconductors due to their excellent electrochemical stability^30^. Fe_2_O_3_ and SnO_2_ have many attractive features, including environmental friendliness, low cost, and natural abundance; particularly, it displays high discharge time at high current density in Li-ion batteries (LIBs)^6,24^.

Recently, studies have been conducted on the electrochemical performance of the active materials of Fe_2_O_3_, SnO_2_ and their composites with other materials as electrodes in supercapacitors. Ardakani et al.^31^ reported a high specific capacitance of 168 F g^-1^ at 5 mV s^-1^ for the α-Fe_2_O_3_@CeO_2_ core–shell heterostructure on a stainless steel substrate in 2 M Na_2_SO_4_ solution, which was prepared by co-precipitation method. Geerthana et al.^32^ synthesized ternary α-Fe_2_O_3_/MnO_2_/rGO heterostructures via a solvothermal method and pasted on the nickel (Ni) foam, which performed the maximum specific capacitance value of 447 F g^-1^ at 1 A g^-1^ in 6 M KOH solution. Cao et al.^33^ synthesized the lignin-based multi-channels carbon nanofibers (MCNFs)@SnO_2_ nanocomposites by the co-electrospinning method, which cast on nickel foam and indicated the high energy storage capacitance of 406 F g^-1^ at 0.5 A g^-1^ in 6 M KOH electrolyte solution. Asaithambi et al.^34^ examined the supercapacitor performance of Ce-SnO_2_@g-C_3_N_4_ composites which deposited on the Ni foam (NF) and reported the high capacitance of 274 F g^-1^ at 0.5 A g^-1^ in 2 M KOH, the active materials prepared by hydrothermal method.

In the present work, we have prepared unique hollow heterostructured materials at various calcination temperatures, including two parts, one is the SnO_2_ hollow nanofibers and the other is the scattered Fe_2_O_3_ hexagonal nanoplatelets on the surface of SnO_2_ hollow fibers. The SnO_2_ hollow nanofibers are the composites’ skeleton that can enhance the surface area and conductivity of the composites. On the other hand, the scattered Fe_2_O_3_ nanoplatelets can be considered muscles that attach to the skeleton and play a supporting role for efficient charge transfer and mass transfer in the charge–discharge process for supercapacitor applications.

## Experimental

### Materials

Tin (II) chloride dihydrate (SnCl_2_·2H_2_O) and potassium hydroxide (KOH) were purchased from Chem-Lab (Belgium). Iron (III) chloride hexahydrate (FeCl_3_·6H_2_O), *N*, *N*-Dimethylformamide (DMF), carbon black, *N*-methyl-2-pyrrolidinone (NMP), polyvinylidene difluoride (PVDF) were obtained from Merck (Germany). Polyvinylpyrrolidone (PVP, 1,300,000 g/mol) and pure ethanol (C_2_H_5_OH, 99.9%) were purchased from Sigma Aldrich and Samchun companies, respectively.

### Preparation of nanofibers

**Figure 1:**
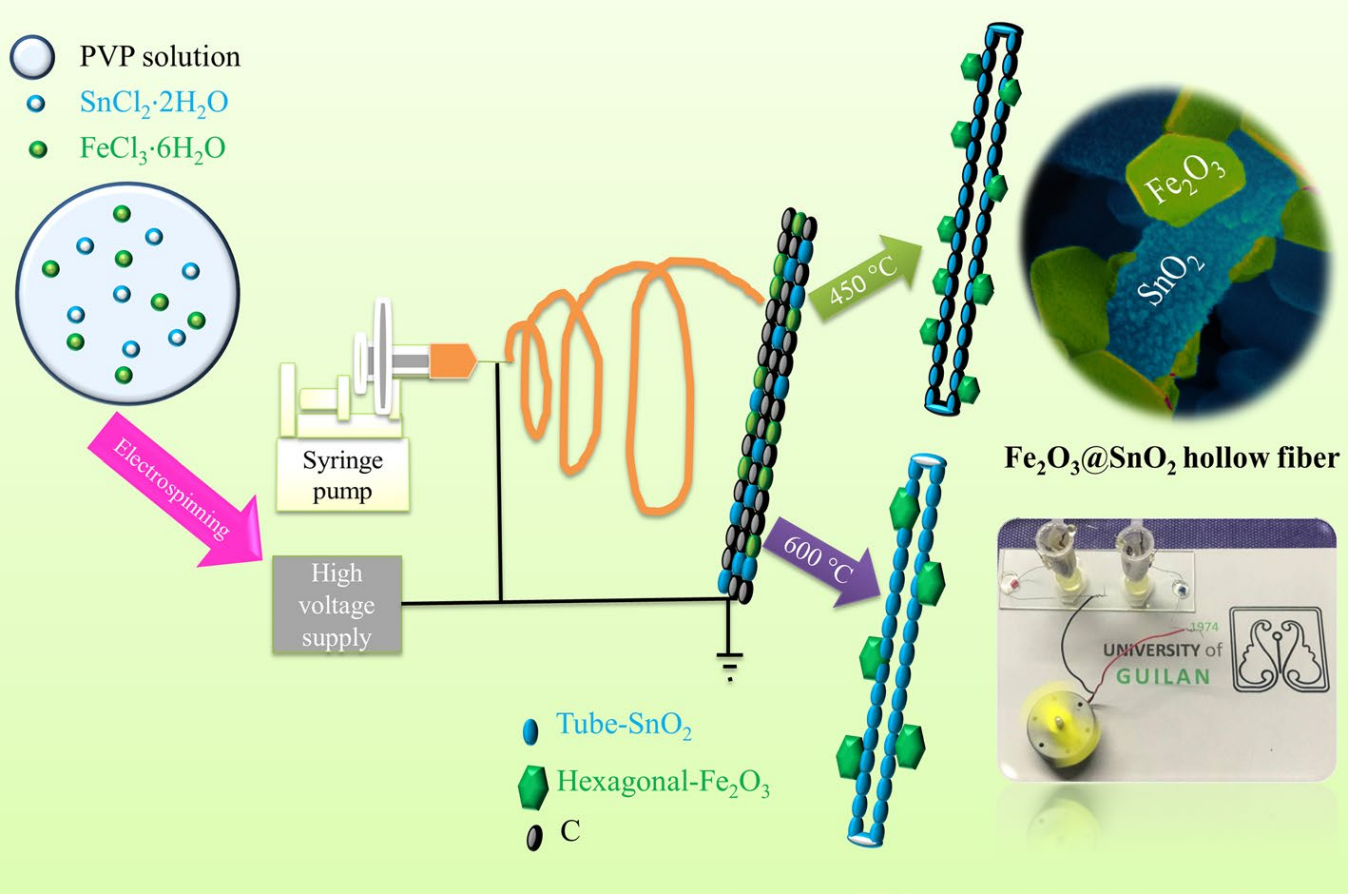
The illustrative scheme for the formation mechanism of Fe_2_O_3_ hexagonal nanoplatelets anchored on SnO_2_ nanofibers and camera-captured photograph of mini fan driven by two asymmetric supercapacitor devices.

The Fe_2_O_3_@SnO_2_ composite nanofibers were prepared by sol–gel electrospinning method at different calcination temperatures. Firstly, 0.81 g FeCl_3_·6H_2_O and 0.34 g SnCl_2_·2H_2_O were stirred in 7.5 ml of ethanol and DMF as solvents for 30 min. Then, 0.6 g PVP was gradually added to the mixture and magnetically stirred overnight. The obtained sol was transferred into 10 ml syringe with a needle of 23G which was fed by a syringe pump at the rate of 0.4 ml h^-1^ and a high voltage of 16.5 kV to produce nanofiber composites. The needle was set at a distance of 10 cm from the aluminum foil collector. The as-spun nanofibers were obtained by drying at 100 °C for 18 h in the oven. Finally, Fe_2_O_3_@SnO_2_–450 and Fe_2_O_3_@SnO_2_–600 composite nanofibers were generated at the calcination temperatures of 450 and 600 °C for 2 h with a ramp rate of 2 °C min^-1^ under an air furnace, respectively. The synthesis schematic is displayed in Fig. 1.

### Characterization

The thermal properties of the as-spun Fe_2_O_3_@SnO_2_ composite were performed by thermogravimetric-derivative thermogravimetry (TGA-DTG) analysis using Bahr model STA 504 thermal analyzer instrument. The structure of the prepared samples was analyzed by X-ray diffraction (X'Pert Pro, Panalytical) using Cu Kα (λ = 1.5406 Å) radiation. Fourier-transform infrared (FTIR) spectra of the samples were recorded by the Bruker Alpha in the range of 400–4000 cm^-1^. Field emission scanning electron microscopy (FESEM) with an energy dispersive spectroscopy (EDS, MIRA3, TESCAN-XMU) was used to investigate the morphology of the mentioned samples. The absorption spectra of the samples were measured by Varian Cary 100 UV/Visible spectrophotometer. The magnetic parameters of the composites were investigated using a vibrating sample magnetometer (VSM, Magnetic Daghigh Kavir Co., Iran) at room temperature (300 K) with a maximum applied magnetic field of ± 15 kOe.

### Electrochemical measurements

The working electrodes were prepared by mixing active materials (Fe_2_O_3_@SnO_2_ composites), carbon black and PVDF as a binder at a weight ratio of 80:10:10 in NMP as a solvent to form a homogeneous slurry. The Ni foam (NF) substrates (2 × 1 cm^2^) were successively cleaned in aqueous HCl (3 M), deionized water, ethanol, and acetone for removing the NiO layer using an ultrasonic device each for 30 min, subsequently dried in the oven at 65 °C for 1 h. The prepared homogeneous slurries were pasted onto nickel foam (1 × 1 cm^2^) and dried overnight at 130 °C. The mass of the active materials on electrodes was about 1 mg. The electrochemical measurements of the electrodes were executed in a three-electrode system by Zahner Zennium device, comprised of Fe_2_O_3_@SnO_2_ composite as a working electrode, platinum wire as a counter electrode and Ag/AgCl as a reference electrode in 3 M KOH electrolyte at room temperature. Cyclic voltammetry (CV) was recorded at a potential window from 0 to 0.5 V, galvanostatic charge–discharge (GCD) was tested in the potential between 0 and 0.43 V, and the electrochemical impedance spectroscopy (EIS) was performed at the frequency ranges from 100 kHz to 10 mHz at an open circuit potential of 0.01 V and 5 mV AC amplitude. The impedance spectra were fitted by the equivalent circuit using the Z-view software.

## Result and discussion

### Thermal analysis

**Figure 2:**
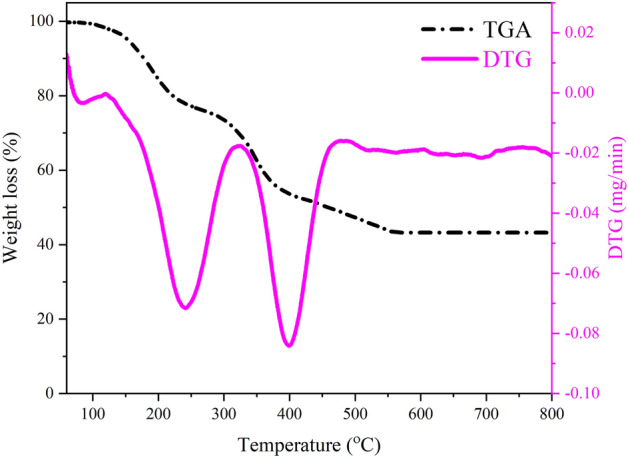
TGA/DTG curves of as-spun Fe_2_O_3_@SnO_2_ nanofibers.

The suitable temperature to decompose PVP, remove of residual compounds and form Fe_2_O_3_@SnO_2_ nanofibers was determined through the thermogravimetric measurement in argon atmosphere from room temperature to 800 °C with a heating rate of 10 °C/min. The TGA and its derivative (DTG) curves for the as-spun nanofibers are shown in Fig. 2, demonstrating the weight loss percentage as a function of temperature. Three distinct stages were observed in weight loss. The first stage with a weight loss of about 12.53% at the range of room temperature to 200 °C could be ascribed to the evaporation of the solvents, including ethanol and DMF in the as-spun nanofibers^35^. The second weight loss in the temperature range of 200–350 °C corresponds to the decomposition of the metal precursors, which is verified by an endothermic peak centered at 240 °C in the DTG curve^36,37^. The third stage is the strong weight loss of 28.42% occurred at around 400 °C, which could be ascribed to the formation of metal oxide phases and the decomposition of the polymer side chain^35^. Above 550 °C in the TGA curve, no change was observed in the mass of the nanofibers, indicating complete combustion of PVP and the formation of crystalline Fe_2_O_3_@SnO_2_ composite.

### XRD analysis

**Figure 3:**
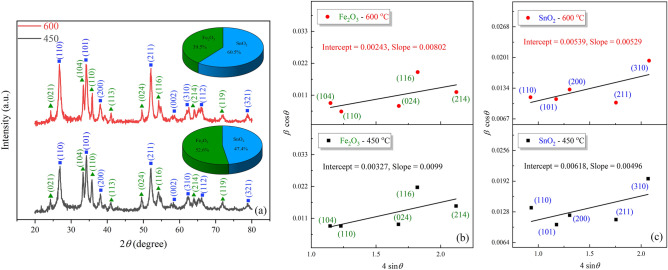
(**a**) XRD patterns (the inset of crystalline phase percentage of Fe_2_O_3_@SnO_2_ heterostructures), W–H plots for (**b**) Fe_2_O_3_ and (**c**) SnO_2_ during calcination.

The X-ray diffraction (XRD) patterns of the calcined samples at 450 and 600 °C are depicted in Fig. 3. The emerged diffraction peaks can be allotted to rhombohedral Fe_2_O_3_ (JCPDS card No. 33-0664, space group: R-3c, 167) and tetragonal SnO_2_ (JCPDS card No. 41-1445, space group: P42/mnm, 136). Also, there were no traces of the other phases in the XRD patterns. The phase fractions of constituent components in the XRD pattern were evaluated by the Rietveld analysis in HighScore plus (Malvern Panalytical) software and obtained values are displayed in Table 1. The crystallite size and strain of the product were calculated from two reliable approaches, the Williamson-Hall (W–H) equation^38^ and the Rietveld refinement analysis using PANalytical X'pert HighScore Plus software^39^. Extracted data from the mentioned analysis revealed that the crystallite size increased and the lattice strain decreased with increasing calcination temperature, respectively. For Fe_2_O_3_@SnO_2_-450 sample, the phase fractions of α-Fe_2_O_3_, and SnO_2_ were estimated to be 52.6 and 47.4%, respectively. Meanwhile, these phases contribute 39.5 and 60.5% to Fe_2_O_3_@SnO_2_-600 sample, respectively. The detailed results are represented in Table 1.

**Table 1:** XRD parameters of Fe_2_O_3_@SnO_2_ nanostructures at various calcination temperatures.

Sample	Constituent phases	Structure	Crystallite size W–H (nm)	Strain (W–H)	Crystallite size Rietveld refinement (nm)	Strain (Rietveld refinement)	Goodness of fit	Phase percentage (%)
Fe_2_O_3_@SnO_2_–450	Hematite: 33–0664	Rhombohedral	42.4	990 × 10^–5^	27.28	175 × 10^–5^	1.58	52.6
	Cassiterite: 41–1445	Tetragonal	22.4	496 × 10^–5^	13.07	130 × 10^–5^		47.4
Fe_2_O_3_@SnO_2_–600	Hematite: 33–0664	Rhombohedral	57.0	802 × 10^–5^	35.98	106 × 10^–5^	1.86	39.5
	Cassiterite: 41–1445	Tetragonal	25.7	529 × 10^–5^	13.83	70 × 10^–5^		60.5

### FTIR analysis

**Figure 4:**
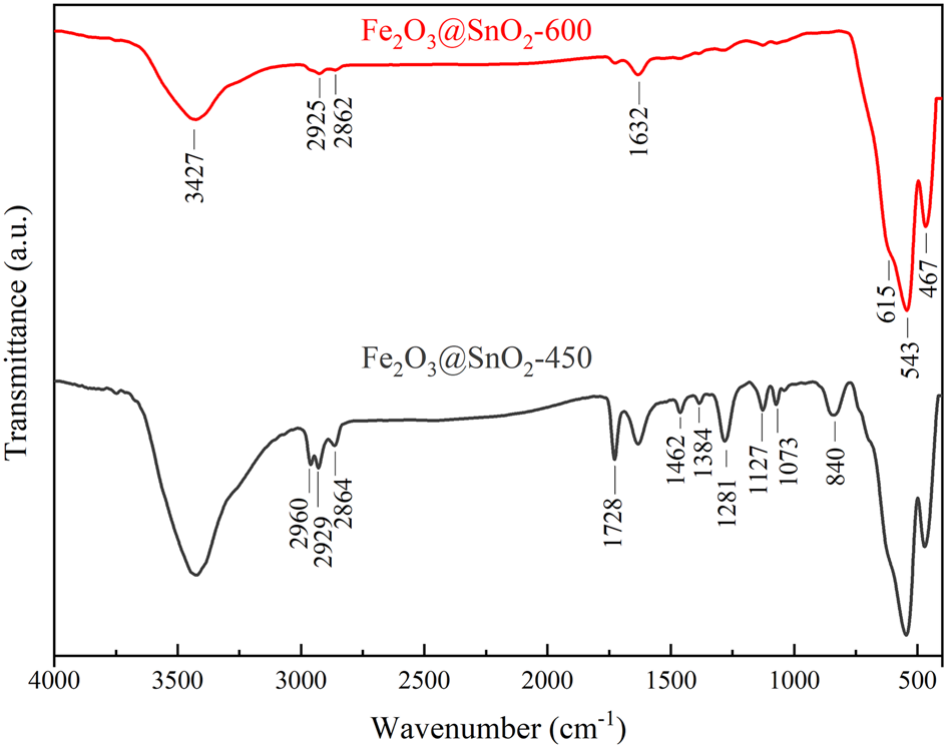
FTIR spectra of Fe_2_O_3_@SnO_2_ at different temperatures.

The FTIR analysis is used to examine the details in the chemical bond structure of the samples which have shown in Fig. 4. The absorption bands under 700 cm^-1^ wavenumber are due to the proximity of O–Sn–O and Fe–O absorption band in the range of 470 cm^-1^; the band at 467 cm^-1^ can be ascribed to overlapped absorption of O–Sn–O and Fe–O^8^. The appeared band at 543 cm^-1^ is allocated to stretching vibration of Fe–O bond and a band that looks like a shoulder at 615 cm^-1^ may be ascribed to stretching vibration of Sn–O bond. In the case of non-metallic bonds, the one that can be observed at 3427 cm^-1^ corresponds to OH stretching vibration of water molecules. The bands which are located around ~ 2960 and 2929 cm^-1^ can be related to asymmetric stretching vibration of CH_2_ and the one that is located around 2864 cm^-1^ can be attributed to symmetric stretching vibration of CH_2_ bond. The two absorptions are seen at 1728 and 1632 cm^-1^ which are assigned to stretch vibrated C = O and that one is observed at 1281 cm^-1^ can be related to asymmetric stretching vibration of C-N. The deformed modes of CH and NCH bonds can be identified around 1462 and 1384 cm^-1^, respectively. Finally, the absorption located at 1127 and 1073 cm^-1^ can be assigned to rocking vibration and at 840 cm^-1^ is ascribed to bending vibration of C–H^40–42^. The absorption intensity of non-metallic bands dramatically decreased with increasing temperature up to 600 °C which can be in agreement with the fact that polymeric compounds such as PVP and DMF almost eliminated at high temperatures.

### Morphological properties

**Figure 5:**
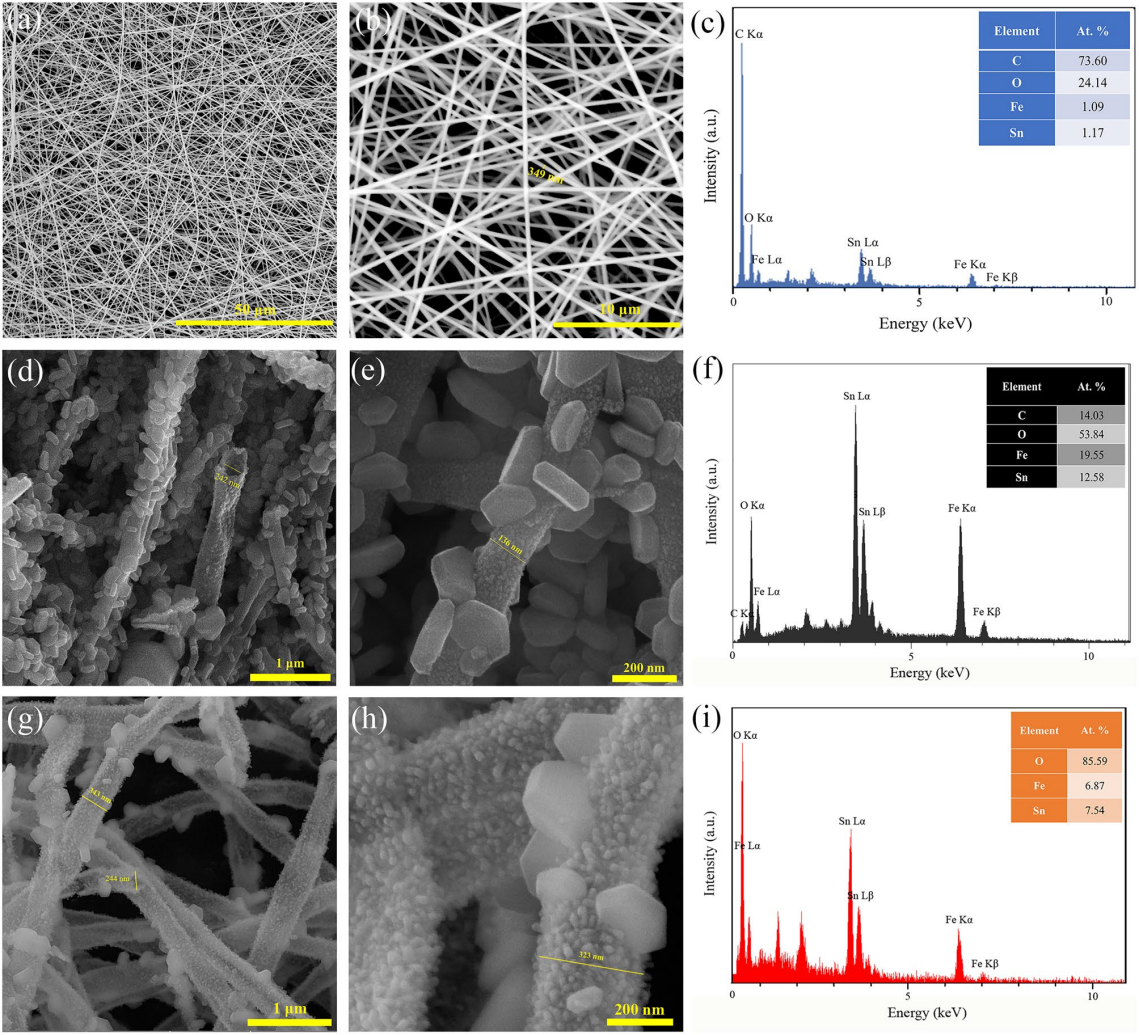
FESEM images for (**a**)–(**c**) as-spun nanofibers, (**d**)–(**f**) Fe_2_O_3_@SnO_2_-450, (**g**)–(**i**) Fe_2_O_3_@SnO_2_-600 and EDS map-scan sum spectra (the inset display the atomic percentage (at%) of the elements).

The FESEM images of the as-spun and calcined Fe_2_O_3_@SnO_2_ nanofibers at different temperatures are revealed in Fig. 5. The as-spun nanofibers (Fig. 5a–c) a smooth and uniform surface with an average diameter of about 349 nm. After the nanofibers were annealed at 450 °C, the average diameter of nanofibers shrunk to 189 nm because of the decomposition of the polymer. The average diameter promotes to 303 nm when the calcined temperature reaches to 600 °C. This phenomenon could be ascribed to the particle growth of the metal oxides^35^. As shown in Fig. 5d–i, the hexagonal plates have grown hierarchically on the hollow nanofibers, which reduce with increasing calcination temperatures from 450 to 600 °C. The Hollow interiors for nanofibers are distinguished in Fig. 5d, g, which is clearly demonstrated that open-ended nanotubes could be maintained during calcination. These open tubular architectures will largely facilitate the ion migration between active material on the electrode surface and electrolyte in the electrochemical process^43^. EDS map-scan sum spectra in Fig. 5c, f, i, revealed the presence of C, O, Fe and Sn elements, which carbon was eliminated with increasing calcination temperature up to 600 °C due to complete thermal decomposition of PVP and metal oxidation.

**Figure 6:**
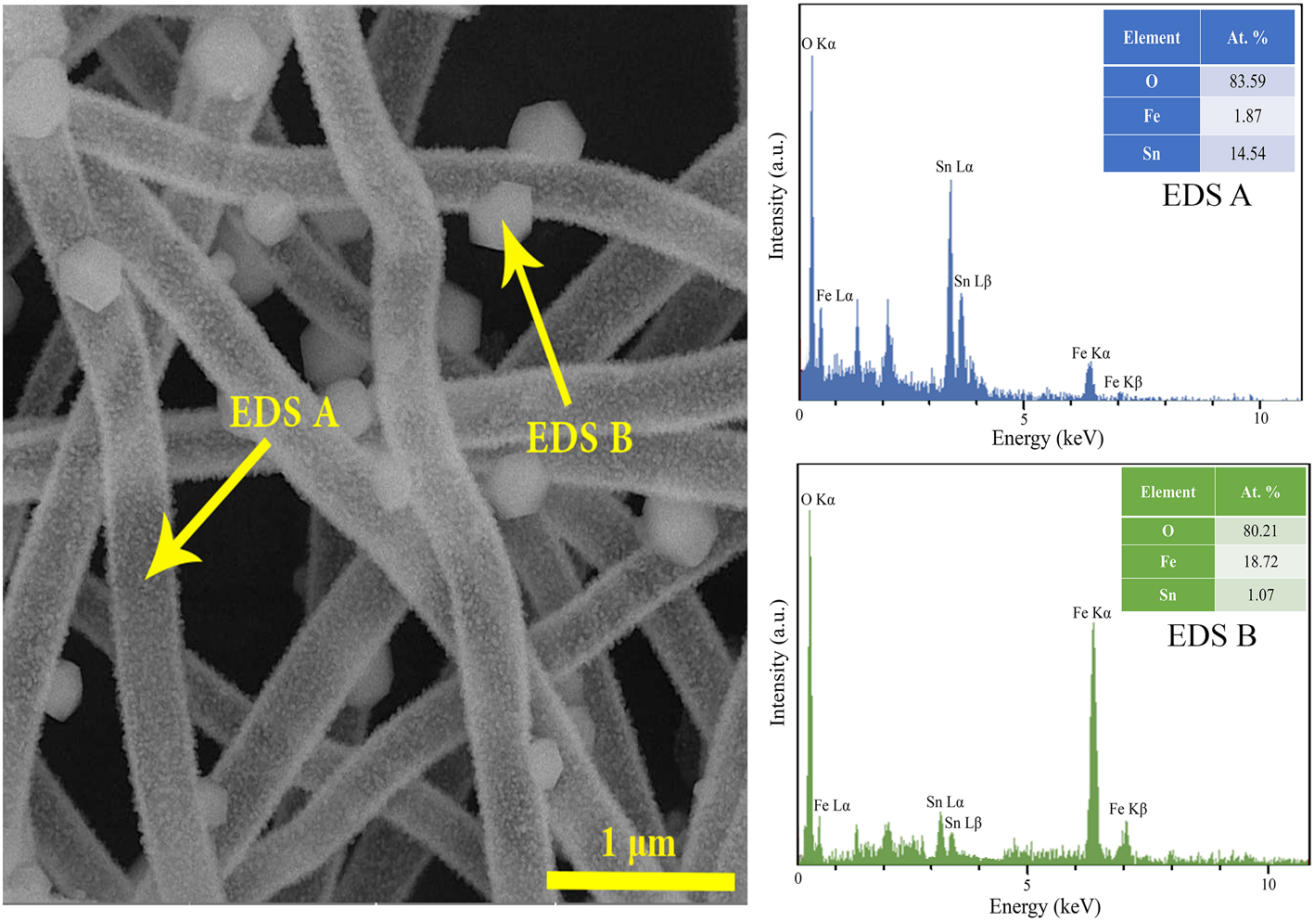
The FESEM image of the Fe_2_O_3_@SnO_2_-600 heterostructure along with the EDS point-scan spectra for the specified regons of A and B.

According to the XRD results, the reduction of the hexagonal platelets on the nanofibers by increasing calcination temperature can be associated with a decrease in the fraction of Fe_2_O_3_ phase, For further clarification of elemental distribution, EDS point-scan analysis of Fe_2_O_3_@SnO_2_-600 composite were recorded in two regions (A and B), which are marked in the FESEM image (Fig. 6). The EDS spectra indicate that Sn and O elements are the dominant constituent elements of nanofibers (Region A) while the content of Fe and O is superior for the hexagonal platelet (Region B). The results prove the formation of Fe_2_O_3_ hexagonal platelets anchored on SnO_2_ nanofibers. Similar morphology was observed for selenization of electrospun carbon nanofibers, including tris(acetylacetonate) iron (III) with polyacrylonitrile (PAN) polymer, at different temperatures under H_2_Se gas, which resulted in the decoration of FeSe nanocrystals on the carbon nanofiber surfaces^44^.

### Optical band gap

**Figure 7:**
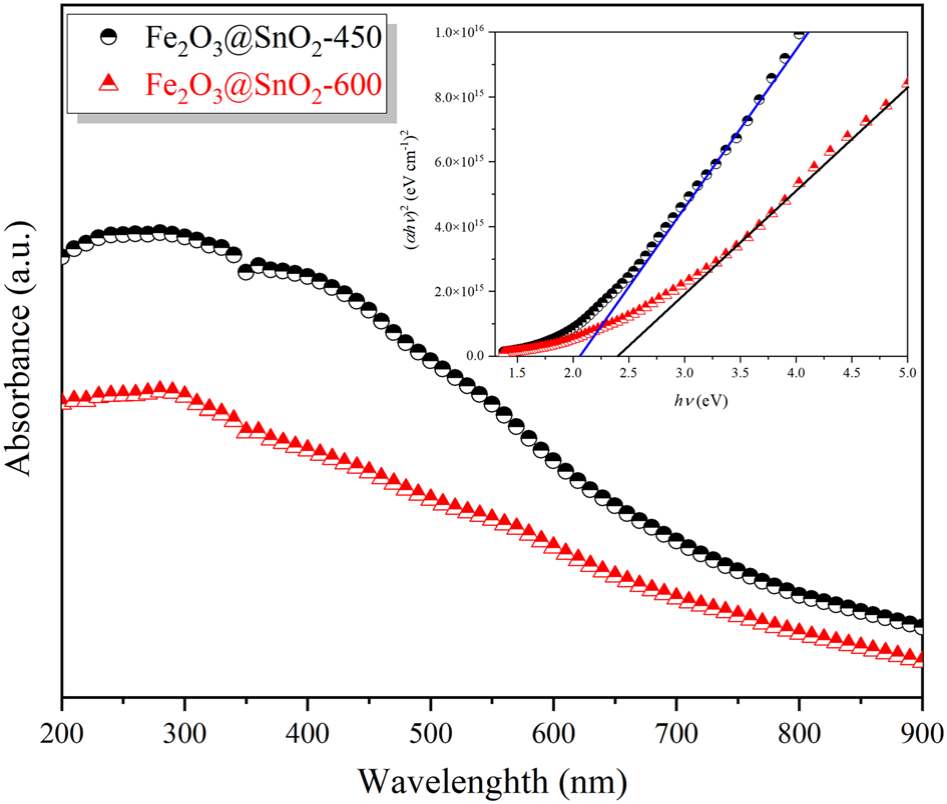
UV–Vis absorption spectra and Tauc plot (inset) for Fe_2_O_3_@SnO_2_ nanocomposites at different calcination tempratures.

UV–Vis absorption analysis was carried out to study of optical properties of the products. Tauc plot was employed to estimate the optical band gap of nanofibers. The optical absorption spectra using Tauc's relation^45^:

1 $$\left( {\alpha h\nu } \right) = A^{*} \left( {h\nu - E_{g} } \right)^{1/2}$$

where A^*^ is a constant, *α* is the absorption coefficient, and h*ν* is the photon energy. The absorption coefficient, *α* was determined from absorption data using the relation^46^:

2 $$\alpha = - \frac{1}{d}ln\frac{{I_{t} }}{{I_{0} }} = \frac{A}{d\log e} \approx 2.303\frac{A}{d}$$

where *d* is the sample thickness which is about equivalent to the quartz cell's path length, and $A = ln\frac{{I_{0} }}{{I_{t} }}$ is the absorbance. The variation of (*α*h*ν*)^2^ vs. photon energy (h*ν*) for relevant composite is shown in the inset of Fig. 7. The direct optical band gap of nanocomposites was calculated via extrapolating the linear part of the (*α*h*ν*)^2^ versus (h*ν*) curve to intercept the energy axis (*α*h*ν* = 0). According to the results by increasing temperature up to 600 °C, the *E*_g_ value was increased from 2.06 to 2.40 eV. This band gap widening can be related to the enhancement of SnO_2_ fraction in composite with an increase in the calcination temperature. The hollow Fe_2_O_3_@SnO_2_-600 nanofiber composite has a lower band gap compared to the *E*_g_ of the bulk SnO_2_ (3.6 eV)^47,48^ and a higher *E*_g_ than the bulk Fe_2_O_3_ band gap (2.2 eV)^20,49^. The deviation of the obtained *E*_g_ from the theoretical values (linear combination of bulk band gaps of constituent phases, SnO_2_ and Fe_2_O_3_) can be related to the structural disorder and surface defects, which cause to the optical band gap narrowing^50^.

### Magnetic properties

**Figure 8:**
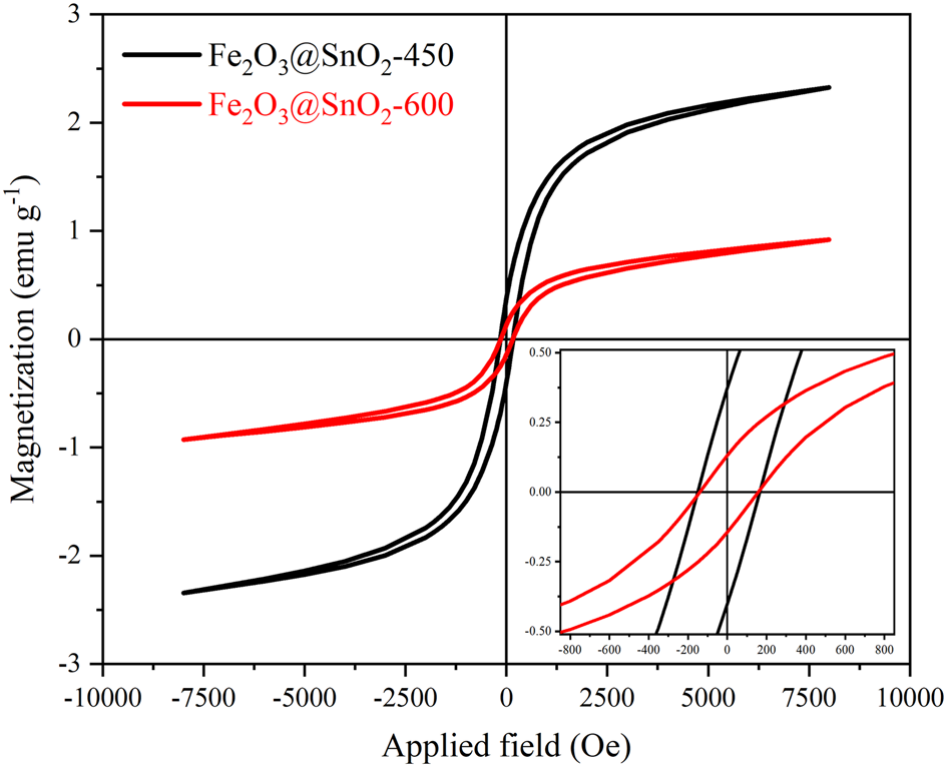
Magnetic hysteresis curve of the hollow Fe_2_O_3_@SnO_2_ nanofibers, the inset shows a magnified view of the curve.

The M–H curves were recorded using VSM analysis between  − 8 to 8 kOe and shown in Fig. 8, confirming the ferromagnetic behavior of the samples. It is clearly seen that the calcination temperature increasing causes the decrease in saturation magnetization (*M*_s_) values of nanofibers from 2.32 to 0.92 emu g^-1^. Both of the samples have very low *M*_s_ compared to similar articles^8,51^. The small *M*_s_ can be ascribed to the diminished effective weight fraction of magnetic core owing to the growth of the non-magnetic SnO_2_ phase in the calcined nanofibers^52^. Furthermore, small changes were observed in the coercive field (*H*_c_) and the remnant magnetization (*M*_r_) of the samples. With calcination temperature increasing the coercive field decreased from 165 to 157 Oe and magnetic remanence decreased from 0.373 to 0.131 emu g^-1^.

### Electrochemical properties

**Figure 9:**
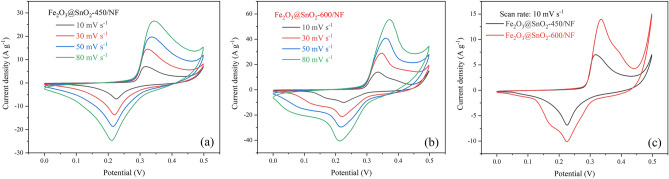
The various scan rates of CV curves for (**a**) Fe_2_O_3_@SnO_2_-450/NF and (**b**) Fe_2_O_3_@SnO_2_-600/NF electrodes. (**c**) Comparison of CV curves of both electrodes at a scan rate of 10 mV s^-1^.

To investigate the electrochemical performance of prepared electrodes, the Fe_2_O_3_@SnO_2_-450/NF and Fe_2_O_3_@SnO_2_-600/NF were tested by a three-electrode system including 3 M KOH electrolyte solution. Figure 9a,b displays the CV curves of both nanofiber electrodes at various scan rates from 10 to 80 mV s^-1^ within the potential window of 0 to 0.5 V. Existence of the two pairs of faradaic redox reaction peaks at the potential of about 0.22/0.33 V is due to the pseudocapacitance behavior of the electrodes in the high scan rates, which implies outstanding electrochemical performance^53^. Furthermore, the pair of redox peaks are nearly symmetrical, which means the high reversibility of the electrodes. The possible redox reactions for Fe_2_O_3_ and SnO_2_ could be explained by the following equations^54,55^:

3 $${\text{Fe}}_{2} {\text{O}}_{3} + 2{\text{e}}^{ - } + 3{\text{H}}_{2} {\text{O}} \leftrightarrow 2{\text{Fe}}\left( {{\text{OH}}} \right)_{2} + 2{\text{OH}}^{ - }$$

4 $${\text{FeOOH}} + {\text{H}}_{2} {\text{O}} + {\text{e}}^{ - } \leftrightarrow {\text{Fe}}\left( {{\text{OH}}} \right)_{2} + {\text{OH}}^{ - }$$

5 $${\text{SnO}}_{2} + 2{\text{e}}^{ - } + {\text{H}}_{2} {\text{O }} \leftrightarrow {\text{SnO}} + 2{\text{OH}}^{ - }$$

The specific capacitance (*C*_s_) for both electrodes from CV curves was calculated by the following Eq. ^56^:

6 $$C_{s} = \frac{\smallint IdV}{{mv{\Delta }V}}$$

where $\smallint IdV$ is the surface area enclosed by the CV curve, $v$ is scan rate (V s^-1^), ${\Delta }V$ is the difference of potential window (V), and *m* is the mass of active materials on the electrodes (g). The *C*_s_ values of Fe_2_O_3_@SnO_2_-450/NF electrode were 299.4, 222.6, 203.8 and 178.8 F g^-1^ at scan rates of 10, 30, 50 and 80 mV s^-1^, respectively. Also, the specific capacitances of Fe_2_O_3_@SnO_2_-600/NF electrode 553.5, 450.1, 394.9 and 330.6 F g^-1^ were achieved at the same scan rates. Increasing the scan rate minimizes the contribution of the electrode's active sites and the diffusion of electrolyte ions on the surface, lowering the *C*_s_ values of the electrodes^57^.

**Figure 10:**
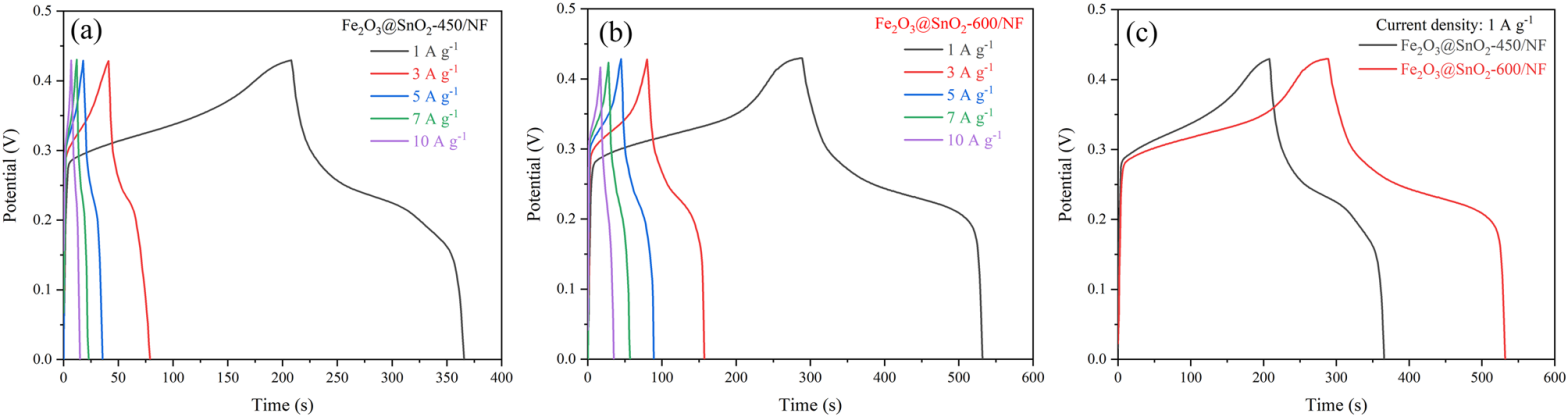
The different current densities of GCD curves for (**a**) Fe_2_O_3_@SnO_2_-450/NF and (**b**) Fe_2_O_3_@SnO_2_-600/NF electrodes. (**c**) Comparison of GCD curves of both electrodes at a current density of 1 A g^-1^.

**Figure 11:**
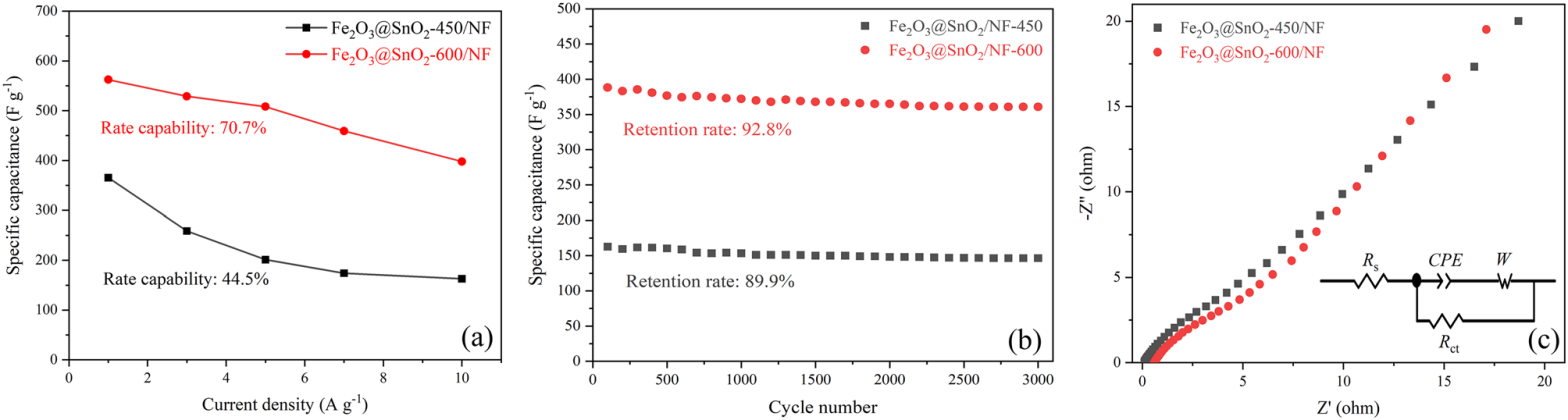
(**a**) The specific capacitances versus current densities, (**b**) cycling stabilities and (**c**) the Nyquist plots with equivalent circuit (inset) of the electrodes.

The GCD curves of both electrodes in the potential range between 0 and 0.43 V at different current densities are depicted in Fig. 10a,b. To avoid the water electrolysis (oxygen evolution reaction) during charging process, a smaller potential window than the CV curve was chosen^58,59^. The pseudocapacitive behavior of the two electrodes was confirmed from potential plateaus in the GCD curves, which correspond to the CV curves in Fig. 9. Also, the IR drop in the GCD curves of both electrodes at the beginning of the discharge time can be due to the internal resistance and energy loss of the electrode materials. The specific capacitance (*C*_s_) is computed from GCD curves using the following equation^56^:

7 $$C_{s} = \frac{I \times \Delta t}{{m \times \Delta V}}$$

where *m* is the mass of active material on the electrode (g), *I* is discharge current (A), $\Delta V$ is the potential window (V), and $\Delta t$ is discharge time (s). The graph of the *C*_s_ values at different current densities for Fe_2_O_3_@SnO_2_-(450 and 600)/NF electrodes is shown in Fig. 11a. The maximum values of *C*_s_ for Fe_2_O_3_@SnO_2_-600/NF electrode were 562.3, 528.8, 508.1, 459.1 and 397.7 F g^-1^ at current densities of 1, 3, 5, 7 and 10 A g^-1^ with 70.7% capability. Also, the specific capacitances for Fe_2_O_3_@SnO_2_-450/NF electrode were 365.3, 258.8, 201.2, 174.2 and 162.8 F g^-1^ at the same current densities with 44.5% capability. The active sites of the electrode at low current densities can appropriately react with electrolyte ions, but at high current densities, the redox reactions only occur on the surface of the active materials due to the limitation of ion diffusion which leads to a decrease of the *C*_s_ values^57^. Figures 9c and 10c are given to compare the CV (at 10 mV s^-1^) and GCD (at 1 A g^-1^) curves for the two electrodes, respectively. As shown in Fig. 11a, with increasing calcination temperature from 450 to 600 °C the specific capacitance is enhanced. It can be due to the reduction of the hematite phase and promotion of the cassiterite phase which leads to more conductivity of the electrode^60–62^. Moreover, Fig. 11b demonstrates the cycling stabilities of Fe_2_O_3_@SnO_2_-450/NF with capacitance retention of 89.9% and Fe_2_O_3_@SnO_2_-600/NF electrode maintained 92.8% of its initial capacitance, which indicates excellent stability of the Fe_2_O_3_@SnO_2_-600/NF electrode. Table 2 compares the values calculated in this work with other reports, including Fe_2_O_3_ and SnO_2_ composites with different materials.

**Table 2:** Comparison of the calculated specific capacitances at a current density of 1 A g^-1^ on Ni foam in this work with other reports.

Active material	Synthesis method	Morphology	Electrolyte	Specific capacitance (F g^-1^)	References
α-Fe_2_O_3_@rGO	Hydrothermal	2D nano-circular-like	2 M KOH	533	^56^
α-Fe_2_O_3_@C	Hydrothermal	Uniform cocoon-like oval spheres	6 M KOH	354.4	^63^
Fe_2_O_3_/MXene	Electrostatic assembly	Urchin-shaped particles on nanosheets	5 M LiCl	486.3	^64^
α-Fe_2_O_3_/MnO_2_/rGO	Solvothermal	Olive-like particles on thin sheet	6 M KOH	447	^32^
Fe-SnO_2_@CeO_2_	Co-precipitation	Spherical grains on the cubic like structure	2 M KOH	348	^65^
Co_3_O_4_@SnO_2_–SnO-250	Hydrothermal	Core–shell	3 M KOH	325	^66^
MCNFs@SnO_2_-5	Co-electrospinning	Multi-channel nanofibers	6 M KOH	298	^33^
Ce-SnO_2_@g-C_3_N_4_	Hydrothermal	Spherical grains on the layer	2 M KOH	274	^34^
Fe_2_O_3_@SnO_2_-600	Sol–gel electrospinning	Hierarchal hexagonal plates on the hollow nanofibers	3 M KOH	562.3	Present work

Electrochemical impedance spectroscopy (EIS) of both electrodes is obtained in a frequency range from 100 kHz to 10 mHz at an open circuit potential, which the Nyquist plots of electrodes are demonstrated in Fig. 11c. All the spectra were fitted using the equivalent circuit (as displayed in the inset of Fig. 11c). The x-axis intercept at high frequencies, the depressed semicircle at high-medium frequencies and the linear line at lower frequencies are assigned to solution resistance (*R*_s_), charge transfer resistance (*R*_ct_) at the electrodes/electrolyte interface and Warburg impedance (*W*), respectively^6,67^. Also, the constant phase element (*CPE*) denotes the double-layer capacitance in simulating the behavior of imperfect dielectrics. The *R*_ct_ value reduced from 10.5 to 9.18 Ω, which demonstrates an increase in the conductivity of the electrodes.

**Figure 12:**
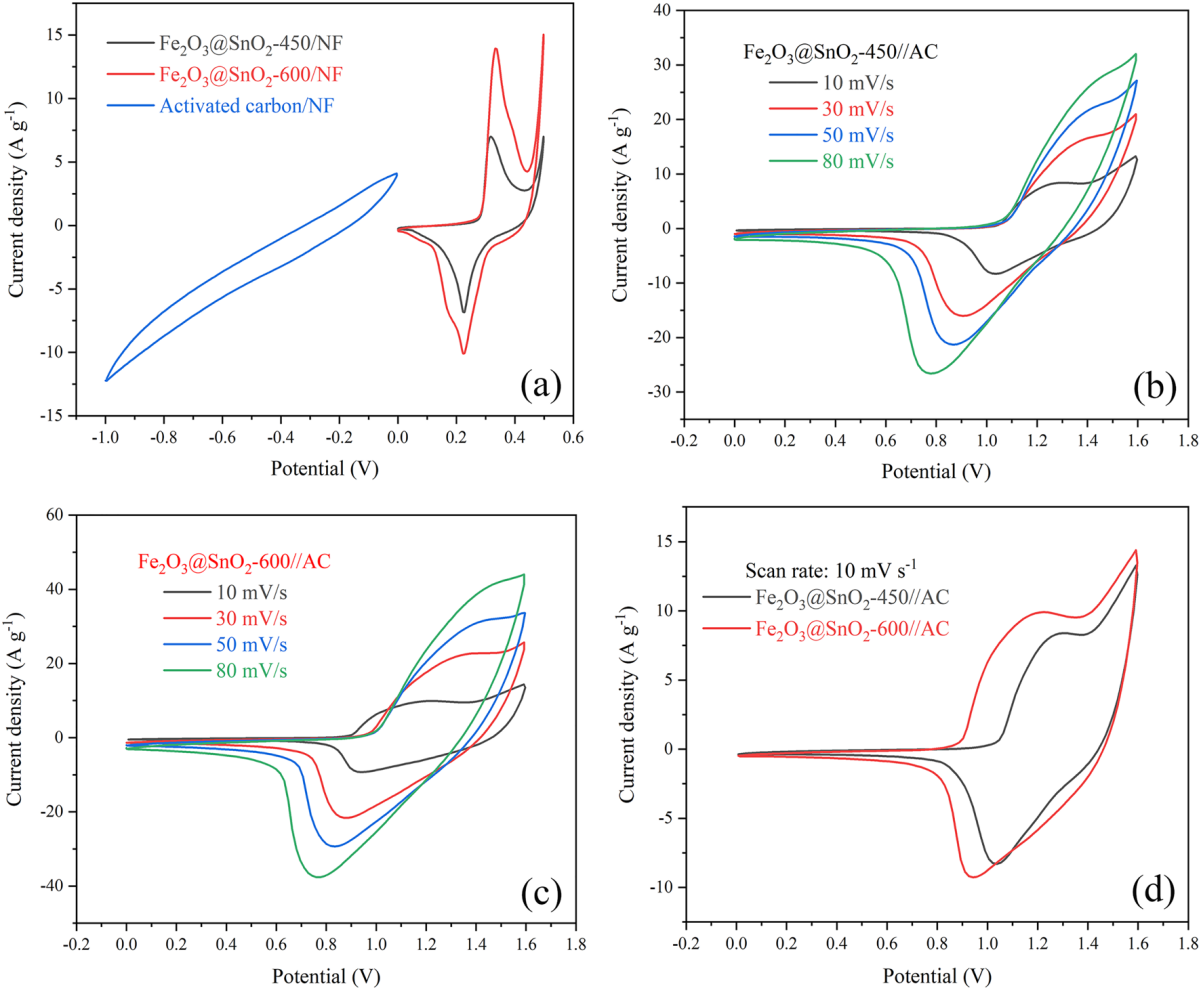
(**a**) CV curve of AC/NF and Fe_2_O_3_@SnO_2_-(450 and 600)/NF at a scan rate of 10 mV s^-1^ in the three-electrode system. (**b**), (**c**) CV curves and (**d**) comparison at 10 mV s^-1^ of Fe_2_O_3_@SnO_2_-(450 and 600)//AC ASC in the two-electrode system.

To evaluate the practical applications of the Fe_2_O_3_@SnO_2_-(450 and 600) nanofiber composites, an asymmetric supercapacitor was built with the electrodes as a cathode, activated carbon pasted on nickel foam (AC/NF) as an anode electrode, and Whatman filter paper as a separator in 3 M KOH electrolyte. Figure 12a illustrates the CV curves of the individual positive electrodes (Fe_2_O_3_@SnO_2_-450 and 600) within a potential range from 0 to 0.5 V and the single negative electrode (AC/NF) from − 1 to 0 V in a three-electrode system at a scan rate of 10 mV s^-1^. To achieve the high electrochemical performance of an asymmetric supercapacitor in a two-electrode system, the charge equilibrium (*Q*_+_  = *Q*_-_) is essential between the two electrodes. Therefore, the mass loading of active materials on the negative and positive electrodes can be inferred by the following equation^68^:

8 $$\frac{{m_{ + } }}{{m_{ - } }} = \frac{{C_{ - } \times \Delta V_{ - } }}{{C_{ + } \times \Delta V_{ + } }}$$

where *C*_+_ (*C*_−_) and Δ*V*_+_ (Δ*V*_−_) are the specific capacitance and working potential window of positive (negative) electrode, respectively. The specific capacitance of positive and negative electrodes can be calculated based on the CV curves using Eq. (6). Figure 12b,c show CV curves of assembled ASCs operated in the voltage range of 0–1.6 V at different scan rates. Figure 12d compares the CV curves for the two ASC devices. The redox peaks represent the pseudocapacitance contributions from the positive electrodes. Also, this pseudocapacitance characteristic is confirmed by GCD curves at various current densities (Fig. 14a,b).

**Figure 13:**
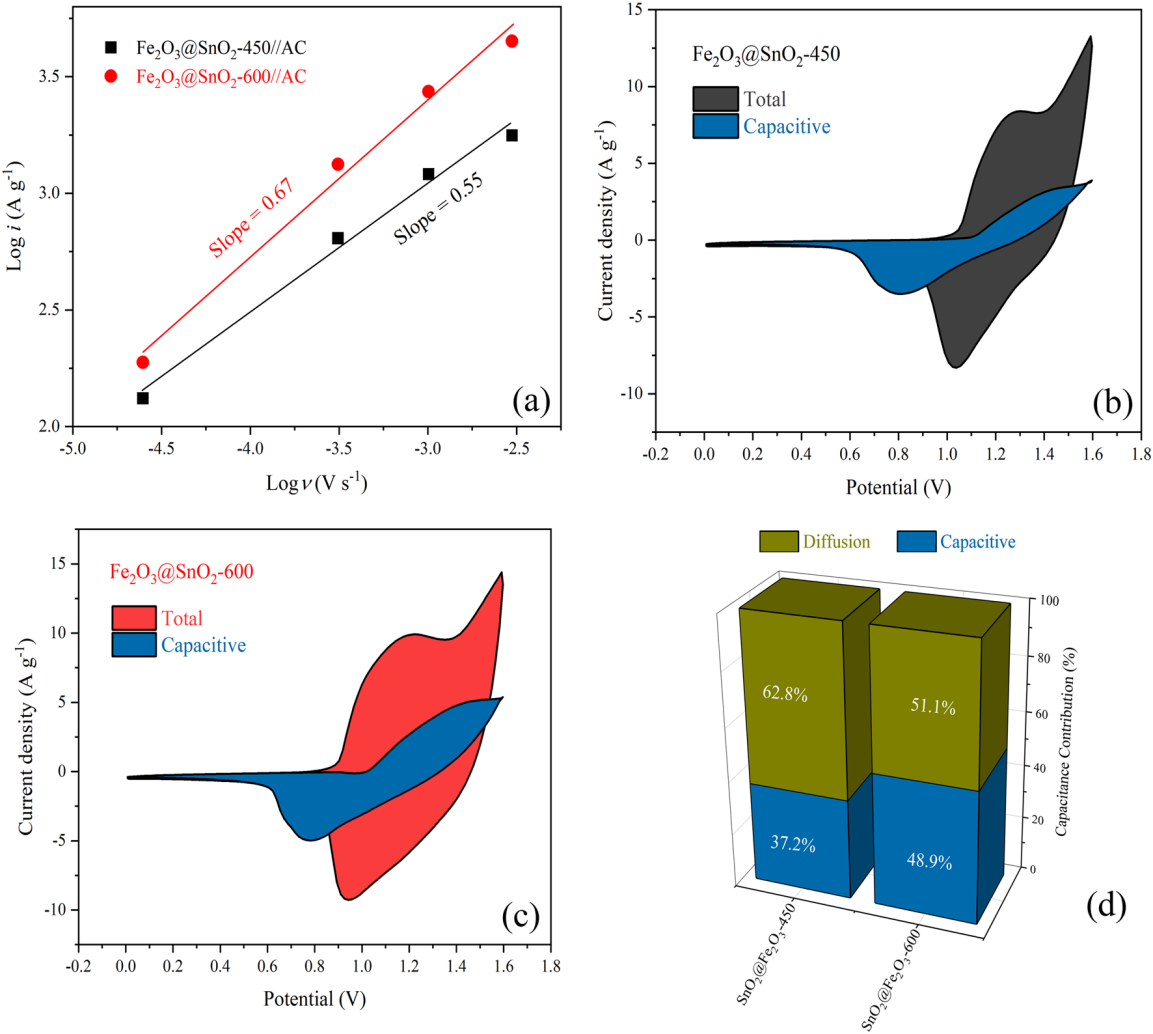
(**a**) The plots of log i versus log ν, the capacitive contribution of CV curves at 10 mV s^-1^ for (**b**) Fe_2_O_3_@SnO_2_-450, (**c**) Fe_2_O_3_@SnO_2_-600. (**d**) Capacitance contribution for two electrodes.

The surface and diffusion controlled charge storage processes could be identified by the power-law relationship^69^:

9 $$i = a\nu^{b}$$

where *i* denotes a current density, *ν* stands for a scan rate, *a* and *b* are adjustable variables. In general, the slope of the plot of log (*i*) versus log (*ν*) at a fixed potential determined the *b*-value. If *b*≈1, the charge storage mechanism is considered the surface-controlled capacitive process, while *b*≈0.5 represents the diffusion-controlled performance. As demonstrated in Fig. 13a, the *b*-values for Fe_2_O_3_@SnO_2_-450 and Fe_2_O_3_@SnO_2_-600 are about 0.55 and 0.67 corresponding to the oxidation reaction peaks, respectively, indicating that the charge storage mechanism is mainly dominated by the ion diffusion-controlled. The contribution of the surface capacitive effect and diffusion-controlled process with the scan rates were determined by Dunn’s equation^69^:

10 $$i\left( V \right) = k_{1} \nu + k_{2} \nu^{1/2}$$

where *i*(V) represents the current response at a given potential, *ν* is a scan rate, *k*_1_ and *k*_2_ are constants. The slope and intercept of the linear relationship between *i*(V)/*ν*^1/2^ versus *ν*^1/2^ give the values of *k*_1_ and *k*_2_, respectively. The shaded blue regions in Fig. 13b,c indicate the surface-controlled contributions at a scan rate of 10 mV s^-1^ for the electrodes, which occupied 37.2 and 48.9% of the total region for the Fe_2_O_3_@SnO_2_-450 and Fe_2_O_3_@SnO_2_-600, respectively (Fig. 13d).

**Figure 14:**
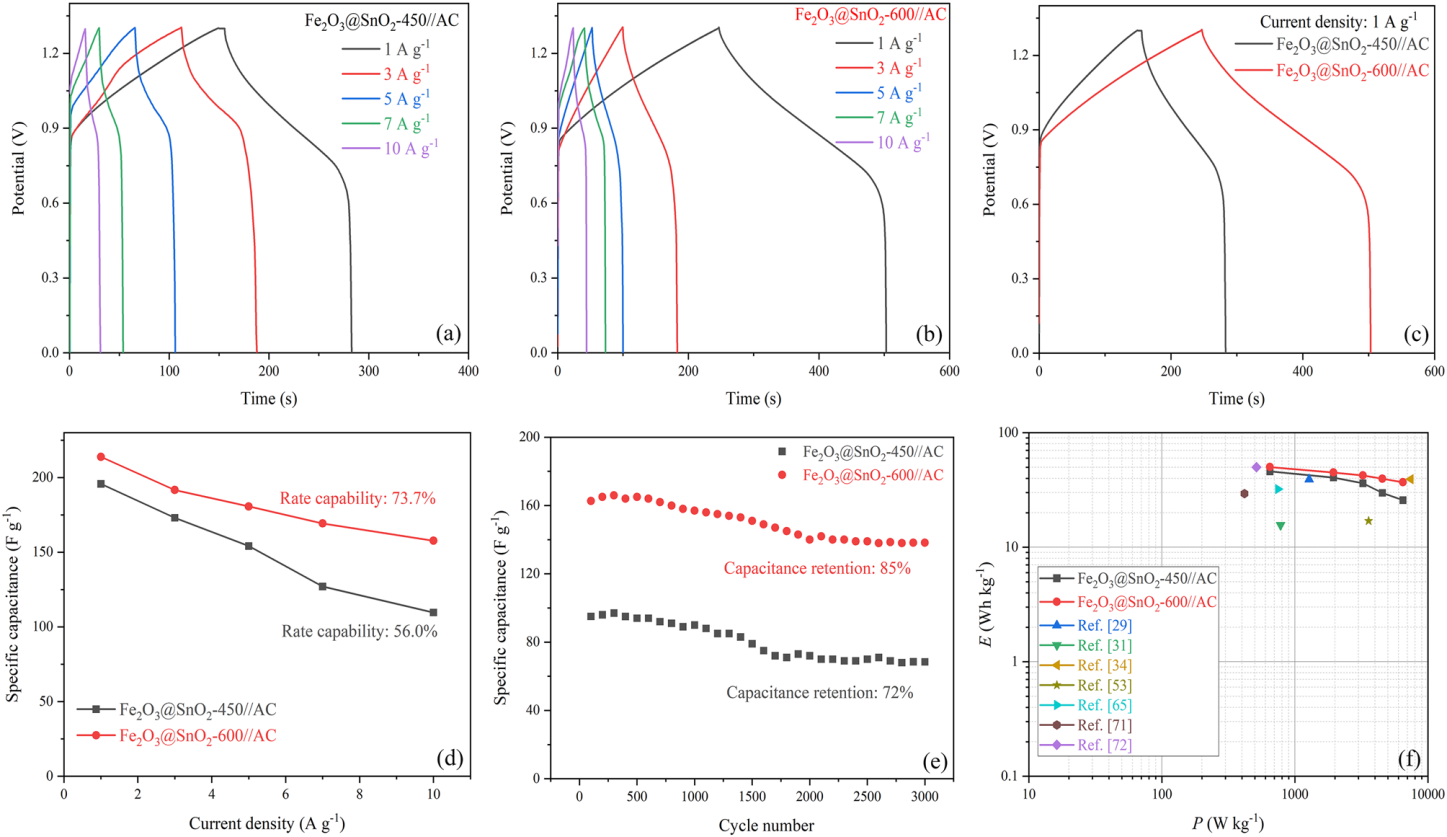
(**a**), (**b**) GCD curves and (**c**) comparison at 1 A g^-1^ of the Fe_2_O_3_@SnO_2_-(450 and 600)//AC. (**d**) Specific capacitances of the ASCs at various current densities. (**e**) Cycling stability performance of the Fe_2_O_3_@SnO_2_-(450 and 600)//AC at a current density of 10 A g^-1^. (**f**) Ragone plot.

According to Eq. (7) and Fig. 14d, the *C*_s_ values of Fe_2_O_3_@SnO_2_-450//AC were obtained of 195.8, 173.1, 154.1, 127.1 and 109.7 F g^-1^ at 1, 3, 5, 7 and 10 A g^-1^, respectively, with 56% rate capability. Also, the maximum *C*_s_ values for Fe_2_O_3_@SnO_2_-600//AC were achieved as 213.9, 191.7, 180.7, 169.3, and 157.7 F g^-1^ at the same current densities with 73.7% rate capability. Figure 14c compares the GCD curves for the two ASC devices.

One of the most critical metrics in asymmetric supercapacitor devices is cycling stability. As shown in Fig. 14e, the cycling stabilities of the Fe_2_O_3_@SnO_2_-(450 and 600)//AC were recorded at a current density of 10 A g^-1^ after 3000 cycles. The ASC devices demonstrate 72 and 85% capacitance retention for Fe_2_O_3_@SnO_2_-450//AC and Fe_2_O_3_@SnO_2_-600//AC. The energy density (*E*_s_) and power density (*P*_s_) are the two main and comparative parameters used to describe the supercapacitor performance. The *E*_s_ (Wh kg^-1^) and *P*_s_ (W kg^-1^) of the Fe_2_O_3_@SnO_2_-(450 and 600)//AC asymmetric supercapacitors are calculated by GCD curves using the following equations^70^:

11 $$E_{{\text{s}}} = \frac{{C_{{\text{s}}} \left( {\Delta V} \right)^{2} }}{7.2}$$

12 $$P_{{\text{s}}} = \frac{{3600 E_{{\text{s}}} }}{\Delta t}$$

The Ragone plot is depicted in Fig. 14f, which relates the energy and power densities of the asymmetric supercapacitors. The maximum *E*_s_ of 45.95 and 50.2 Wh kg^-1^ are achieved at a *P*_s_ of 650 W kg^-1^, as well as the minimum energy densities of 25.7 and 37 Wh kg^-1^ are retained at a higher *P*_s_ of 6500 W kg^-1^ for the Fe_2_O_3_@SnO_2_-450//AC and Fe_2_O_3_@SnO_2_-600//AC ASCs, respectively. These values are superior than most of other reported ASC devices, such as V_O_-Fe_2_O_3_@Sn_2_O_3_ (39.1 Wh kg^-1^ at 1280 W kg^-1^)^29^, RGO||α-Fe_2_O_3_@CeO_2_ (15.62 Wh kg^-1^ at 781 W kg^-1^)^31^, Ce-SnO_2_/g-C_3_N_4_//Activated Carbon (39.3 Wh kg^-1^ at 7425 W kg^-1^)^34^, α-Fe_2_O_3_/SnO_2_/rGO (17 Wh kg^-1^ at 3585 W kg^-1^)^53^, Fe-SnO_2_@CeO_2_ (32.2 Wh kg^-1^ at 747 W kg^-1^)^65^, SnO_2_@C (29.4 Wh kg^-1^ at 418 W kg^-1^)^71^, CC-Fe_2_O_3_/Na_2_WO_4_ NF (50 Wh kg^-1^ at 514.28 W kg^-1^)^72^. The results obtained from Fig. 14 indicate that the Fe_2_O_3_@SnO_2_-600//AC is the most suitable option for ASC device fabrication due to its high specific capacitance and long cycling stability.

**Figure 15:**
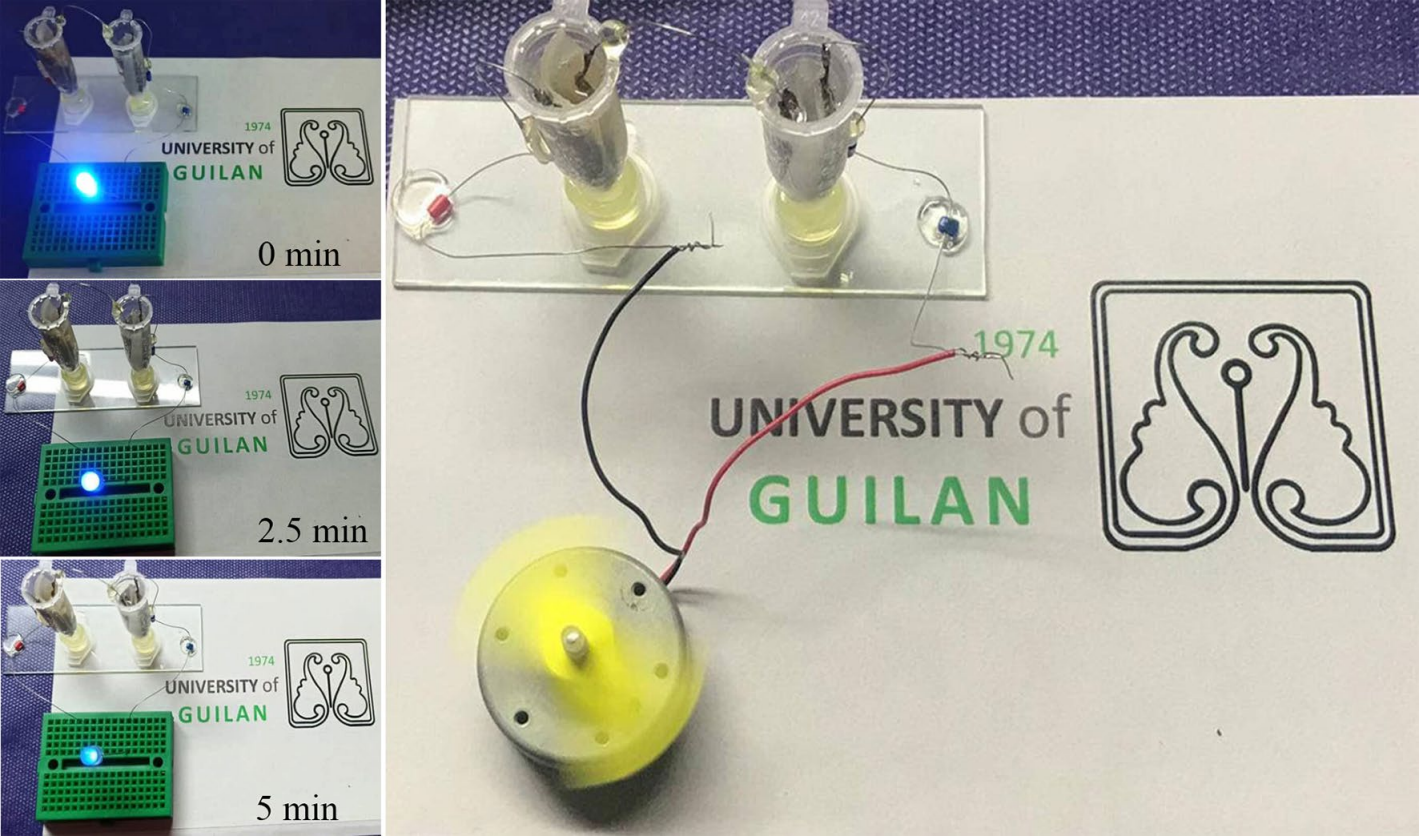
Photograph of Fe_2_O_3_@SnO_2_-600//AC ASC device connected in series powering the blue LED at different times and mini fan.

The best electrode Fe_2_O_3_@SnO_2_-600 was examined as a power source. As shown in Fig. 15, two cells of the ASC device were connected in series and were able to light up the blue light-emitting diode (LED, 20 mA, 3.6 V) for about 5 min after charging by a power supply. In addition, a mini fan (0.1 W, 3 V) can be rapidly rotated by these cells for about 20 s (see Video S1). From the results, it was revealed that the Fe_2_O_3_@SnO_2_-600//AC ASC device had an outstanding performance in storing energy.

## Conclusion

The hollow Fe_2_O_3_@SnO_2_ nanofiber composites were successfully synthesized by the sol–gel electrospinning process at different calcination temperatures of 450 and 600 °C. The composite structures of rhombohedral and tetragonal were confirmed for hematite and cassiterite by XRD analysis, respectively. The phase percentage of SnO_2_ was increased from 47.4 to 60.5% during calcination. FESEM images showed that the hexagonal nanoplatelets of Fe_2_O_3_ are hierarchically anchored on the SnO_2_ hollow nanofibers, which are reduced during calcination from 450 to 600 °C and verified with XRD and EDS analyses. Increasing the cassiterite phase with calcination temperatures grew the optical band gap from 2.06 to 2.40 eV due to the nature of the SnO_2_ band gap. VSM results demonstrated that a significant drop in the saturation magnetization from 2.32 to 0.92 emu g^-1^ during calcination temperatures was due to the reduction of the Fe_2_O_3_ phase. The electrochemical performance of the Fe_2_O_3_@SnO_2_-450 and 600 active materials pasted on the Ni foams indicated that the prepared Fe_2_O_3_@SnO_2_-600/NF electrode has a maximum specific capacitance of 562.3 F g^-1^ at a current density of 1 A g^-1^, a remarkable rate capability (70.7%) and excellent retention (92.8%) after 3000 cycles. Increasing the capacitance contribution from 37.2 to 48.9% during calcination distinguishes the Fe_2_O_3_@SnO_2_-600/NF electrode from another electrode. Furthermore, the assembled Fe_2_O_3_@SnO_2_-600//AC ASC device delivers a maximum energy density of 50.2 Wh kg^-1^ at a power density of 650 W kg^-1^. Overall, this study provides a promising strategy for the production of new hollow nanofiber electrode materials that encounter high power and energy density provisions for supercapacitor applications. The hexagonal platelets Fe_2_O_3_ decorated on SnO_2_ hollow nanofiber is an admirable candidate for electrode material in electrochemical energy storage devices.

## Supplementary Information

Supplementary Video 1.

## Data Availability

All data generated or analyzed during this study are included in this published article, and the datasets used/ or analyzed during the current study are available from the corresponding author on reasonable request.
